# How Foods and Beverages Are Promoted Online: A Content Analysis of the Digital Food Environment in China

**DOI:** 10.3390/nu15245067

**Published:** 2023-12-11

**Authors:** Juan Chen, Yuetong Du, Jian Raymond Rui

**Affiliations:** Center for Public Health Risk Surveillance and Information Communication in Guangdong Province, South China University of Technology, 382 Waihuan East Rd, Guangzhou 510006, China; chenjuan@scut.edu.cn (J.C.); yuetong.du.scut@gmail.com (Y.D.)

**Keywords:** digital food environment, food promotion, content analysis, audience feedback

## Abstract

Digital platforms such as social media and e-commerce platforms have become a major space where foods and beverages (F&B) are promoted. Prior research has found that online, unhealthy F&B receive more presence than healthy F&B. This obesogenic food environment may increase the obesity rate. Therefore, it is critical to understand how healthy and unhealthy F&B are promoted online. A content analysis of 2906 posts related to F&B via five digital platforms was conducted in China, where the obesity rate has increased in recent years. Firstly, the results show that unhealthy F&B received more presence on digital platforms than healthy F&B. Secondly, healthy F&B posts tended to highlight the healthiness of the products, whereas unhealthy F&B posts leveraged a wide range of promotional strategies, specifically use cues, food cues, chewing sounds, sensory descriptions, friend cues, local cultural appeal, nostalgia appeal, price information, discount information, and trending hashtags or topics. Next, use cues, chewing sounds, sensory descriptions, family cues, and friend cues increased the quantity of audience feedback, whereas price information and using trending hashtags or topics lowered the quantity of audience feedback. Moreover, local cultural appeal and social proof exhibited the opposite impact on audience feedback. Finally, health benefit statements lowered audience feedback for healthy F&B posts, whereas brand visibility and purchase links inhibited audience feedback for unhealthy F&B posts. In addition to describing the digital food environment in China, the present research provides implications on how to promote healthy F&B. Particularly, we suggest that healthy F&B businesses and healthy eating campaigns should leverage the strategies unhealthy F&B use to receive more consumer attention, in order to increase their own products’ public visibility and attractiveness.

## 1. Introduction

As a serious public health concern worldwide, obesity is related to a wide range of chronic diseases, which account for over 70% of mortality each year [1]. Although the obesity rate in China is traditionally below that of Western countries [2], this number increased from 3.1% in 2004 to 8.1% in 2018 [3].

One critical factor that contributes significantly to obesity is the food environment, which is defined as the interface where people interact with the wider food system, including external domains (i.e., food availability, prices, vendor and product properties, marketing and regulation) and personal domains (i.e., food accessibility, affordability, convenience, desirability) [4]. Earlier studies on the food environment focused on the built environment (e.g., the density of high-calorie catering near schools or communities), the food industry (e.g., the increasing production of industrialized food), and food marketing on traditional mass media [5,6,7,8,9,10,11]. As Internet-based new media have become an important part of the contemporary media landscape, the role that digital platforms such as social media and e-commerce platforms is playing in constructing the food environment cannot be dismissed [12]. In particular, while traditional mass media tend to feature branded food and beverages (F&B) produced and promoted by commercial companies, Internet-based new media allow ordinary users to share posts of F&B that they have bought and cooked. Consequently, ordinary users can become social media influencers, which can have a significant impact on others’ food choices [13,14]. However, the online food environment seems to have remained unchanged, as research has still found that across the world, digital platforms are more likely to feature F&B that are higher in fat, sugar, and salt (HFSS) than healthy F&B [15,16,17,18,19,20,21].

In China, the prevalence of mobile payments and express deliveries has made F&B highly accessible to consumers online. Thus, the digital food environment may have a critical impact on Chinese adolescents, which can potentially facilitate the obesity problem of this population. However, research on which F&B are promoted through digital platforms in China is scarce. As traditional cuisines are relatively low in fat and sugar [22], we first sought to determine if, like in other countries [15,16,17,18,19,20,21], unhealthy F&B are receiving more digital presence in China than healthy F&B. Notably, we focused on digital platforms that Chinese teenagers often use to search for food-related information, although other age groups may also like these platforms.

RQ1: Are unhealthy F&B featured more via China’s digital platforms than healthy F&B?

In addition, research has explicated what strategies are used to promote F&B. For example, the scholarship on media psychology reveals that commercials tend to employ multisensory cues, such as visual and audio techniques, to seek consumers’ attention [23,24,25,26]. These cues can activate one’s food-related memory and make the featured products more attractive [27]. In addition, cues that demonstrate the eating behavior of multiple persons were also found to increase one’s intention to consume the food [25,28,29,30,31] because social norms are a significant predictor of eating [32,33]. Other strategies were also identified, such as price promotion [34], interactions with audiences [16,35] and healthy food labels [36].

Furthermore, prior research suggests that healthy and unhealthy F&B may use different promotional strategies. For instance, visual cues featuring the eating behavior, which can show the food is palatable, were used more often for unhealthy foods [26]. Moreover, price promotion [34] and community interactions [17,35] were often used by unhealthy foods, whereas healthy food labels [36] and social norm cues [37] were often leveraged by healthy foods.

Although these studies provide insights on how healthy and unhealthy F&B are promoted in the digital environment, these studies were mostly conducted in Western countries [16,17,18,19,23,24,25,26,27,28,29,31], and culture may have a critical impact on F&B promotion. For instance, food may be promoted by enhancing its connection with local culture, history, and tourism [38]. In addition, prior research offers extensive evidence on what strategies could elevate one’s intention to purchase F&B [23,24,25,26,27,28,29,30,31,32,33]. However, this intention is not necessarily the same as audience feedback such as liking, commenting on, and sharing food-related posts. Hence, the promotional strategies that can facilitate audience feedback still remain unknown.

RQ2: What strategies are used to promote unhealthy and healthy F&B? Is there any significant difference in terms of the promotional strategies that unhealthy and healthy F&B leverage?

RQ3: How are these promotional strategies related to audience feedback for unhealthy and healthy F&B?

Through a content analysis approach, this study sought to answer two additional questions above. The results are expected to find additional F&B promotional strategies and provide practical implications on promoting healthy eating in China.

## 2. Materials and Methods

### 2.1. Sampling

#### 2.1.1. The Population of Interest

This study is part of a larger project that aims to understand the relationship between digital platform usage and childhood obesity in China. We focused on Chinese teenagers aged between 11 and 16 for the following considerations. Firstly, children that are too young may not have enough pocket money to purchase food independently from their parents. Thus, it is their caregivers who make food choices. Moreover, many high schools in China require students to live in dorms on campus. These adolescents tend to have enough money for food and receive limited parental intervention on their usage of digital platforms. Consequently, although they have not reached adulthood, their behaviors regarding digital platform usage and food choices can be similar to those of young adults.

Taken together, our population of interest should not be too young or too old. In China, ten is considered a milestone in one’s childhood. Once they are over 10 years old, children are expected to take more responsibilities, and parents also allow more independence for them. Additionally, as there can be large regional differences in terms of the age when children enter the school system, most children should finish middle school and start high school at 16 years old. Hence, 11–16 is the age range of our target population.

#### 2.1.2. Digital Platforms

Prior to the content analysis, we conducted interviews to understand what digital platforms our target population used to search for food-related information. A total of 28 interviews were conducted. Five digital platforms were mentioned the most: Bilibili, Douyin, Kuaishou, Xiaohongshu, and Pinduoduo.

These five platforms vary in their major affordances and target users. Douyin, Kuaishou, and Bilibili provide similar affordances that enable users to share videos. However, Bilibili allows for longer videos, whereas videos via Douyin and Kuaishou are usually limited to two minutes. Moreover, Kuaishou is targeted at residents of relatively low socioeconomic statuses and rural areas, whereas Douyin is targeted at residents of large cities.

Xiaongshu affords a wider range of media content sharing, including text-based messages, photos, and videos. Notably, commercials are allowed, and purchase links are made available via all four platforms mentioned above.

Finally, Pinduoduo is an e-commerce platform similar to Amazon. Most product information is presented as text and images, with fewer videos. As the current content analysis only involved the data from publicly available Internet services, institutional approval was not required.

#### 2.1.3. Data Collection

We collected F&B content data from these platforms through web crawling techniques between January 2022 to February 2023. Given the differences between the platforms, we adjusted our data collection. As there is a food section via Bilibili and Pinduoduo, we searched food-related posts in this section. Specifically, we accessed 14,436 videos through Bilibili. We deleted similar videos and employed a stratified random sampling. Specifically, we calculated the percentages of the five categories of videos in the food section of Bilibili (i.e., cooking, taste test, food exploration, picnics, and live records of food). Next, we randomly selected videos from these categories and adjusted their numbers based on their percentages. In total, 600 videos were included for formal coding.

As for Pinduoduo, we accessed the top 600 food-related posts by employing MobDuos data analysis software [39]. After deleting duplicate and irrelevant posts, 500 were kept for formal coding.

The other three platforms do not have a food section, so we used different data collection techniques. Specifically, we accessed 12,880 videos via Kuaishou using 45 food-related hashtags, which were selected through an exhaustive search by two graduate students. Again, we calculated the percentages of these hashtags and randomly selected 606 videos for formal coding based on the percentages of these hashtags.

The data collection methods used for Douyin and Xiaohongshu were similar. Following two major social media indices in China [40,41], we accessed the top 100 food-related influencers on Douyin and Xiaohongshu. Then, we randomly selected six posts from each influencer, leading to 600 posts for each platform. Therefore, the final sample size was 2906.

Notably, as our goal is to conduct a systematic investigation on the digital food environment in China, we did not distinguish the types of food-related posts. Any posts about the food were sampled, whether offered by commercial companies or ordinary users.

### 2.2. Coding Scheme

#### 2.2.1. Basic Information

The first part of the coding scheme involves the basic information of F&B posts, which includes the platform and the category of F&B. We operationalized unhealthy F&B as the F&B that are high in fat, sugar, or salt (i.e., HFSS foods), following the guidance for less healthy food [42]. Specifically, this guidance provides a list of categories of food considered as HFSS. Thus, F&B that were not on this list were coded as healthy.

#### 2.2.2. Audience Feedback

In response to RQ3, we recorded several metrics indicating how audiences react to F&B online. As different platforms use different metrics, we used three indices shared by Bilibili, Kuaishou, Douyin, and Xiaohongshu: likes, favorites, and comments. Since Pinduoduo does not have any available data about audience feedback, we had to exclude Pinduoduo from this measurement.

#### 2.2.3. Promotional Strategies

We built the coding scheme of the promotional strategies upon prior research, which examined how F&B are promoted. According to these studies, we built several first-level categories, including (1) food cues and eating-related sensory experiences, (2) social influences, (3) health- and nutrition-based qualities, and (4) price-related information. After the preliminary coding, we designed second-level categories and added new categories, which became the final coding scheme presented in Table 1.

It is common to use multisensory cues in commercials to increase the appeal of F&B to audiences [23,24,25,26]. Following this research, we included visual presentation and chewing sounds in our coding scheme. Visual presentation includes two subcategories: use cues and food cues. While *use cues* refer to whether eating behavior was present in the post [25], *food cues* refer to whether the food was present in the post. Chewing sounds was conceptualized as the sound of chewing the food or drinking the beverage, and operationalized as whether the sound was present in the post. In addition, we added *sensory description*, conceptualized as verbal or textual descriptions of the sensory characteristics of the F&B, and operationalized as whether such a description was present in the post.

Following research on social influences on eating [29,30,31,32], we included three types of social cues—*family cues*, *friend cues*, and *social proof*. These former two were defined as whether family and friends were present in the F&B post. In addition, social proof was operationalized as whether there was a statement indicating the support of the product from certain groups.

As another commonly used food marketing strategy [43], *nostalgia appeal* was conceptualized as using nostalgic retro scenes or elements to evoke memories of the old days. We operationalized this as whether such scenes and elements were present in the post.

*Cultural appeal* was a new category that we added to the coding scheme, based on our preliminary coding. We identified two subcategories of cultural appeal: historical appeal and local cultural appeal. *Historical appeal* refers to whether the F&B was linked to a historical or cultural event, story, or festival. *Local cultural appeal* refers to whether the F&B was associated with a place.

Fuchs et al. (2022) found that healthy F&B often used health labels for marketing and promotions [36]. In the present study, we identified three ways commercials use to demonstrate that F&B are healthy and of high quality. *Quality description* was conceptualized as statements about the quality of the ingredients or the production procedure. A *health benefit statement* was conceptualized as any statement about the health-enhancing benefits such as prevention of disease, improving fitness, and not gaining weight. A *nutritional statement* was conceptualized as a description of the nutrition of the F&B and operationalized as whether such statements are present in the post.

Following Bennett et al. (2020), we recognized price promotion as an important marketing strategy for F&B and identified two subcategories [34]. While *price information* was operationalized as whether the price of the product was mentioned in the post, *discount information* was operationalized as whether the post mentioned discounts, limited time offers, gifts, or cashback rewards.

In addition, we identified three other methods used to promote F&B products and sales throughout our preliminary coding. *Brand visibility* was operationalized as whether the information that indicated the product brand was present in the post such as the brand name, logo, iconic packaging, or slogan. *Availability of purchase links* was operationalized as whether the post mentioned a link to purchase the product. *Use of trending hashtags or topics* was operationalized as whether a trending hashtag or topic was mentioned in the post to attract public attention.

### 2.3. Coding Process

Ten trained graduate assistants were responsible for the coding, with two assistants for each platform. First, two coders for each platform took 20% of the contents and coded this part of the data independently. Then, we calculated Krippendorff’s alpha values as the intercoder reliabilities between the two coders of each platform for each category, and all reliability tests exceeding 0.8 indicate good intercoder reliability. Afterwards, two coders from each platform split the remaining F&B posts in the sample and each coded half of them.

### 2.4. Data Analysis

Data were analyzed via SPSS 24. Descriptive analysis was run to provide proportion data for themes of F&B content and the healthiness of F&B presented in the data (RQ1). Chi-square analysis was employed to compare the promotional strategies used by healthy and unhealthy F&B (RQ2). In response to RQ3, we conducted the Mann–Whitney U test, as the audience feedback indices were not normally distributed (likes: min = 0, max = 1,660,000, mean = 67,806.20, skewness = 4.73, kurtosis = 28.06; favorites: min = 0, max = 873,000, mean = 13,344.28, skewness = 9.59, kurtosis = 136.37; comments: min = 0, max = 139,000, mean = 2798.74, skewness = 6.71, kurtosis = 60.37).

## 3. Results

### 3.1. Healthiness of China’s Digital Food Environment (RQ1)

RQ1 asks if unhealthy F&B received more coverage on China’s digital platforms than healthy F&B. Descriptive statistics revealed that 71% of F&B posts featured unhealthy F&B. Bilibili (78.8%) had the largest proportion of unhealthy F&B posts, followed by Xiaohongshu (76.8%), Douyin (72.0%), Kuaishou (66.2%), and Pinduoduo (59.4%). Therefore, the current digital food environment in China has a tendency for covering unhealthy F&B.

### 3.2. Promotional Strategies Used by Healthy and Unhealthy F&B (RQ2)

RQ2 asked whether there are significant differences between healthy and unhealthy F&B in terms of the promotional strategies they used. The chi-square tests revealed that unhealthy F&B posts were more likely to employ use cues (*χ*^2^(1) = 67.45, *p* < 0.001; see Table 2), food cues (*χ*^2^(1) = 7.80, *p* = 0.005), chewing sounds (*χ*^2^(1) = 71.33, *p* < 0.001), sensory descriptions (*χ*^2^(1) = 5.02, *p* = 0.025), friend cues (*χ*^2^(1) = 11.25, *p* = 0.001), local cultural appeal (*χ*^2^(1) = 7.31, *p* = 0.007), nostalgia appeal (*χ*^2^(1) = 23.47, *p* < 0.001), price information (*χ*^2^(1) = 21.62, *p* < 0.001), discount information (*χ*^2^(1) = 5.41, *p* = 0.020), and trending hashtags or topics (*χ*^2^(1) = 5.92, *p* = 0.015) than healthy F&B. In contrast, healthy F&B posts were more likely to use quality descriptions (*χ*^2^(1) = 63.40, *p* < 0.001), health benefits statements (*χ*^2^(1) = 204.70, *p* < 0.001), and nutritional statements (*χ*^2^(1) = 18.04, *p* < 0.001) than unhealthy F&B.

### 3.3. Effect of Promotional Strategies on Audience Feedback (RQ3)

RQ3 asked which promotion strategies were related to audience feedback that healthy and unhealth F&B posts received. We conducted the Mann–Whitney U test to answer this question. Overall, unhealthy F&B posts received more likes (*U* = 501,210.50, *p* < 0.001), favorites (*U* = 524,846.50, *p* = 0.008), and comments (*U* = 497,758.00, *p* < 0.001) than healthy F&B. We present our findings for the four categories below.

#### 3.3.1. Strategies That Affected Audience Feedback for Healthy F&B

Table 3 shows the effects of promotional strategies on audience feedback for healthy F&B. The following strategies were found to facilitate audience feedback for healthy F&B: *use cues* (like: *U* = 33,577.00, *p* < 0.001; favorites: *U* = 43,188.50, *p* = 0.001; comments: *U* = 30,316.00, *p* < 0.001); *chewing sounds* (like: *U* = 29,596.00, *p* < 0.001; favorites: *U* = 36,362.50, *p* < 0.001; comments: *U* = 28,760.00, *p* < 0.001); *sensory descriptions* (like: *U* = 26,733.50, *p* < 0.001; favorites: *U* = 24,669.50, *p* < 0.001; comments: *U* = 27,747.00, *p* < 0.001); *family cues* (like: *U* = 16,711.00, *p* < 0.001; favorites: *U* = 18,923.00, *p* = 0.018; comments: *U* = 16,992.00, *p* < 0.001); and *friend cues* (like: *U* = 10,298.50, *p* < 0.001; favorites: *U* = 12,282.50, *p* = 0.020; comments: *U* = 10,138.50, *p* < 0.001). These strategies were positively associated with the numbers of likes, favorites, and comments for healthy F&B.

Next, *price information* was negatively related to the numbers of likes (*U* = 17,841.00, *p* = 0.040) and favorites (*U* = 15,862.50, *p* = 0.001) for healthy F&B posts. *Using trending hashtags or topics* also lowered the number of likes (*U* = 33,258.00, *p* < 0.001), favorites (*U* = 36,292.50, *p* = 0.014), and comments (*U* = 32,559.00, *p* < 0.001) for healthy F&B posts.

Thirdly, *local cultural appeal* increased the numbers of likes (*U* = 16,839.00, *p* < 0.001) and comments (*U* = 16,523.00, *p* < 0.001) for healthy F&B posts. Similarly, *social proof* reduced the numbers of likes (*U* = 11,444.50, *p* = 0.026) and comments (*U* = 11,372.00, *p* = 0.022) for healthy F&B posts.

Finally, *health benefit statements* lowered the numbers of likes (*U* = 17,422.50, *p* < 0.001), favorites (*U* = 19,910.00, *p* < 0.001), and comments (*U* = 18,276.50, *p* < 0.001) for healthy F&B posts.

#### 3.3.2. Strategies That Affected Audience Feedback for Unhealthy F&B

Table 4 shows the effects of promotional strategies on audience feedback for unhealthy F&B. Similarly to healthy F&B, *use cues* (like: *U* = 256,758.50, *p* < 0.001; favorites: *U* = 327,686.00, *p* < 0.001; comments: *U* = 230,324.00, *p* < 0.001); *chewing sounds* (like: *U* = 288,107.50, *p* < 0.001; favorites: *U* = 334,771.00, *p* < 0.001; comments: *U* = 267,728.00, *p* < 0.001); *sensory descriptions* (like: *U* = 183,912.50, *p* < 0.001; favorites: *U* = 184,472.00, *p* < 0.001; comments: *U* = 187488.00, *p* < 0.001); *family cues* (like: *U* = 131,273.50, *p* < 0.001; favorites: *U* = 132,970.50, *p* < 0.001; comments: *U* = 136,425.00, *p* < 0.001); and *friend cues* (like: *U* = 143,795.50, *p* < 0.001; favorites: *U* = 153,024.00, *p* < 0.001; comments: U = 159,257.00, *p* = 0.001) elevated the numbers of likes, favorites, and comments of posts featuring unhealthy F&B.

Next, *price information* lowered the numbers of likes (*U* = 200,406.00, *p* < 0.001), favorites (*U* = 180,912.50, *p* < 0.001), and comments (*U* = 208,584.50, *p* < 0.001) for unhealthy F&B posts. *Using trending hashtags or topics* was negatively related to the numbers of likes (*U* = 300,202.50, *p* = 0.011) and comments (*U* = 284,869.50, *p* < 0.001) received by unhealthy F&B posts.

Thirdly, *local cultural appeal* lowered the number of favorites received by unhealthy F&B posts (*U* = 194,256.00, *p* < 0.001). *Social proof* was positively related to the numbers of likes (*U* = 82,911.00, *p* = 0.032), favorites (*U* = 83,933.50, *p* = 0.051), and comments (*U* = 83,566.00, *p* = 0.043) for unhealthy F&B posts.

Finally, *brand visibility* (like: *U* = 329,316.50, *p* = 0.029; favorites: *U* = 328,291.00, *p* = 0.023; comments: *U* = 327,682.50, *p* = 0.019) and *purchase links* (like: *U* = 190,381.00, *p* = 0.001; favorites: *U* = 176,951.50, *p* < 0.001; comments: *U* = 194,750.00, *p* = 0.006) were negatively associated with the numbers of likes, favorites, and comments received by unhealthy F&B posts.

## 4. Discussion

### 4.1. Principal Findings

Media has become an important component of contemporary society; it exhibits a great impact on what we eat. Thus, it is critical to understand how digital platforms construct our food environment. Through a quantitative content analysis of the F&B posts on five digital platforms in China, we revealed how healthy and unhealthy F&B in China are promoted online. These strategies provide important implications on how to promote healthy eating.

Consistent with other countries [15,16,17,18,19,20,21], we found a serious unhealthy tendency in China’s digital food environment. Moreover, as our investigation is not limited to only one platform, our results revealed the nuanced differences in the F&B posts across different platforms. Specifically, unhealthy F&B were more prevalent on social media such as Bilibili and Xiaohongshu, compared to Pinduoduo. Therefore, although individuals can find healthy and unhealthy F&B via e-commerce platforms, they are more likely exposed to unhealthy F&B through social media. Given the prevalence of social media, the consequences of this unhealthy F&B trend can be serious. Thus, we advocate that healthy F&B should be given more presence and visibility online, which we will elaborate upon later.

Next, our study compared the promotional strategies used by healthy and unhealthy F&B posts. We found that healthy F&B posts were more likely to use quality descriptions, health benefit statements, and nutritional statements to highlight their healthiness. This suggests that healthy F&B businesses consider healthiness as the primary advantage of their product. In contrast, unhealthy F&B posts leverage a more diverse range of promotional strategies than healthy ones, including multisensory cues, social cues (specifically friend cues), local cultural appeal, nostalgia appeal, and price promotions. This contrasting promotional strategy may explain why unhealthy F&B received more likes, favorites, and comments than healthy F&B. Indeed, healthy food commercials tend to emphasize the health benefits of their products rather than the taste [26]. However, consumers may be more concerned with whether the food is tasty. Thus, emphasizing the healthiness rather than the tastiness of food could reduce consumers’ interest in healthy food commercials and lower their intention to purchase these products [26]. Thus, we advocate that healthy eating campaigns or commercials should use more of the same promotional strategies that unhealthy F&B marketing uses.

Furthermore, we analyzed what promotional strategies affected audience feedback indices for healthy and unhealthy F&B posts. Our investigation revealed several important findings. Firstly, multisensory cues, specifically use cues, chewing sounds, and sensory descriptions, enhanced audience feedback for both types of posts. This finding aligns with prior research that demonstrated that multisensory cues elevate audience attention to the food and their intention to consume the food [23,24,25,26]. Specifically, use cues can activate an audience’s memory about eating similar foods [27] and trigger cravings for the food [25]. Prior research focuses primarily on the visual images of use cues and sensory cues [23,24,25,26]. Our study extends this research by suggesting that textual descriptions (i.e., sensory descriptions) and auditory presentations of sensory experiences (i.e., chewing sounds) may also enhance one’s interest in the food, which future research can test.

Secondly, two types of social cues—family cues and friend cues—were found to encourage audience feedback. Prior research found that social cues function as a type of social norm that can elevate one’s intention to purchase food [25,32,44,45]. Our study provides indirect support to these studies.

One unexpected finding is that social proof facilitated audience feedback for unhealthy F&B posts but inhibited audience feedback for healthy F&B posts. One possible explanation is that people may be aware of the threat of unhealthy F&B. Thus, they need validations for their consumption of these F&B, which social proof cues can offer. In contrast, social proof cues in healthy F&B posts may be interpreted as reminders of a healthy diet, which can make people feel pressured. Consequently, this can trigger psychological reactance, which lowers their interest in the product [46]. This suggests that there is a boundary condition for the social proof technique in terms of persuasion.

Additionally, we found several promotional strategies that exhibited negative impacts on audience feedback for healthy or unhealthy F&B posts. These strategies include trending hashtags or topics, price information, discount information, brand visibility, availability of purchase links, and health benefit statements. These negative impacts may result because these strategies are commonly used for marketing. Therefore, individuals may associate them with persuasive intentions, which could cause psychological reactance [46], and thereby lower their interest in the product.

### 4.2. Theoretical Implications

Our study provides following theoretical implications for the research on food promotional strategies. Firstly, we added to the extant scholarship that focused on food promotional in the Western world by providing several new strategies, specifically local cultural appeal and historical appeal. These may be related to China’s rich history and cultural traditions.

Secondly, we found five strategies that could facilitate audience feedback for both healthy and unhealthy F&B, specifically use cues, chewing sounds, sensory descriptions, family cues, and friend cues. These strategies were found to increase food attractiveness to consumers [23,24,25,26,27,28,29,30]. Our findings extend the effect of these strategies to audience feedback on digital platforms. Furthermore, as explained earlier, our results suggest that auditory and textual descriptions of eating experiences may also elevate one’s interest in the food.

Finally, our results found several strategies that can backfire. Although empirical evidence is lacking, we provide plausible explanations that link these strategies to psychological reactance. These explanations offer directions for future research.

### 4.3. Practical Implications

This study offers several practical implications for health campaigns and public health policies in China. Firstly, given the unhealthy trend in China’s digital food environment, unhealthy F&B should be required to inform consumers of possible health risks that may be derived from their products. Currently, F&B companies are required to provide nutritional information on the product package. However, this information is often invisible in many commercials and social media posts or livestreaming. Therefore, we suggest that legislation should require unhealthy F&B businesses to provide nutritional information in their online presentations, and that this information should be easy to read.

In addition, promoting healthy F&B should be encouraged on digital platforms. For example, the platform should improve their algorithms so that healthy F&B can achieve more presence. Incentives can also be offered to users that share healthy F&B.

Furthermore, healthy F&B businesses and healthy eating campaigns should leverage the promotional strategies that have helped unhealthy F&B gain consumer attention. For instance, as suggested earlier, healthy F&B posts may benefit from use cues, chewing sounds, and sensory descriptions to trigger consumers’ craving for the product. Moreover, using images of family and friends may also improve persuasion outcomes for healthy F&B marketing. Multiple cues can be used together to maximize the persuasive effect. For example, after teaching how to finish a healthy meal, the character in the video could show that they are eating the meal together with family or friends (use cues, friend cues, and family cues), describe how flavorful it is (sensory description), followed by a recording of the crispy sounds of the food being eaten (chewing sounds).

### 4.4. Limitations and Future Directions

This study has several limitations. Firstly, although our selection of digital platforms was based on interviews with Chinese teenagers, the interview sample was rather small and not representative. This can introduce bias into our findings.

Secondly, as mentioned earlier, Pinduoduo did not make audience feedback indices publicly available, so we could not measure the effectiveness of promotional strategies on this platform. Thus, our discussion of the impact of promotional strategies is limited to social media platforms. Future research can collaborate with e-commerce platforms to access sales data and examine how these promotional strategies are related to product sales.

Thirdly, it is important to note that Pinduoduo may essentially be different from other platforms. In addition to being an e-commerce platform that aims to sell F&B, Pinduoduo usually allows for textual and visual presentations. In contrast, a large portion of the posts through other platforms are videos. Although we included Pinduoduo based on the interview results, caution is still required when results between these platforms are compared.

Additionally, our sample was limited to almost 3000 posts from five digital platforms in China. This obviously limited the internal and external validity of our results. Future research should replicate our analysis with a random sample that includes more posts from more digital platforms. Cross-cultural comparisons may also provide valuable insights on how healthy and unhealthy F&B are promoted differently between different countries.

Furthermore, the current study did not examine how these promotional strategies were related to the eating behaviors or intentions of subjects. Future research needs to consider conducting experiments to test whether certain promotional strategies can affect the intentions or behaviors of eating.

## 5. Conclusions

The present study employed a content analysis approach to examining how healthy and unhealthy foods are presented on five popular digital platforms in China. In addition to finding that unhealthy foods receive more presence online, our results reveal that unhealthy foods leveraged a wider range of promotional strategies than healthy foods, which tend to emphasize their healthiness. Moreover, promotional strategies that could facilitate audience feedback for posts about healthy and unhealthy foods were identified. Thus, healthy food companies may need to learn from unhealthy food companies about how to better promote themselves online.

## Figures and Tables

**Table 1:** The finalized coding scheme.

Category		Conceptualization	Operationalization
**Basic information**			
Platform		The digital platform where food and beverage-related content was published.	Bilibili = 1; Douyin = 2; Kuaishou = 3; Pinduoduo = 4; Xiaohongshu = 5
Healthiness of the F&B		Whether the food or the beverage presented in the post is healthy or unhealthy; unhealthy F&B are the F&B that meet at least one of the HFSS food criteria.	Healthy = 1; Unhealthy = 2
**Audience feedback**			
Likes		The number of likes received by each post.	**/**
Favorites		How many times each post was added to one’s favorites or personal collection.	**/**
Comments		The number of comments received on each post.	**/**
**Promotional strategies**			
Visual presentation	Use Cue	A scene of eating the food or drinking the beverage.	Absence = 0; Presence = 1
	Food Cue	An image of the food or the beverage.	Absence = 0; Presence = 1
Chewing sounds ^a^		Sounds of chewing the food or drinking the beverage.	Absence = 0; Presence = 1
Sensory description		A verbal or textual description of the sensory characteristics of the food.	Absence = 0; Presence = 1
Family cue		An image of family.	Absence = 0; Presence = 1
Friend cue		An image of friends.	Absence = 0; Presence = 1
Social proof		A statement indicating the support of the product from certain groups.	Absence = 0; Presence = 1
Nostalgia appeal		Using nostalgic retro scenes or elements to evoke memories of the old days.	Absence = 0; Presence = 1
Cultural appeal	Historical appeal	The post is linked to a historical or cultural event, story, or festival.	Absence = 0; Presence = 1
	Local cultural appeal	The food is associated with a place.	Absence = 0; Presence = 1
Quality description		A statement about the quality of the ingredient or the production procedure.	Absence = 0; Presence = 1
Health benefits statement		A statement about the health-enhancing benefits such as prevention of disease, improving fitness, and not gaining weight.	Absence = 0; Presence = 1
Nutritional statement		A description of the nutrition of the F&B.	Absence = 0; Presence = 1
Price promotion ^b^	Price information	The price of the product.	Absence = 0; Presence = 1
	Discount information	Discounts, limited time offers, gifts, or cashback rewards.	Absence = 0; Presence = 1
Brand visibility ^b^		Information that indicates the product brand.	Absence = 0; Presence = 1
Availability of purchase links ^b^		A link to purchase the product.	Absence = 0; Presence = 1
Use of trending hashtags or topics		A trending hashtag or topic (e.g., best-selling food online, latest product, upcoming festival)	Absence = 0; Presence = 1

^a^ Content presented as text and images were excluded from the coding of this category. ^b^ Pinduoduo was excluded from the coding of this category since Pinduoduo is an e-commercial platform that inherently shows prices, discounts, and advertisements.

**Table 2:** Differences between promotional strategies used by healthy and unhealthy F&B (*n* = 2906).

Category ^a^	Healthy F&B, *n* (%)	Unhealthy F&B, *n* (%)	*χ* ^2^	*p*	All, *n* (% Total)
Use cue	320 (38.0)	1131 (54.8)	67.45	<0.001	1451 (49.9)
Food Cue	826 (98.1)	2049 (99.3)	7.80	0.005	2875 (98.9)
Chewing sounds ^b^	237 (33.6)	988 (52.2)	71.33	<0.001	1225 (47.2)
Sensory description	656 (77.9)	1683 (81.5)	5.02	0.025	2339 (80.5)
Family cue	122 (14.5)	252 (12.2)	2.77	0.096	374 (12.9)
Friend cue	76 (9.0)	279 (13.5)	11.25	0.001	355 (12.2)
Social proof	55 (6.5)	123 (6.0)	0.34	0.559	178 (6.1)
Nostalgia appeal	11 (1.3)	108 (5.2)	23.47	<0.001	119 (4.1)
Historical appeal	8 (1.0)	14 (0.7)	0.59	0.443	22 (0.8)
Local culture appeal	107 (12.7)	345 (16.7)	7.31	0.007	452 (15.6)
Quality description	253 (30.0)	348 (16.9)	63.40	<0.001	601 (20.7)
Health benefit statement	204 (24.2)	120 (5.8)	204.70	<0.001	324 (11.1)
Nutritional statement	106 (12.6)	157 (7.6)	18.04	<0.001	263 (9.1)
Price information ^c^	74 (11.6)	349 (19.8)	21.62	<0.001	423 (17.6)
Discount information ^c^	12 (1.9)	67 (3.8)	5.41	0.020	79 (3.3)
Brand visibility ^c^	95 (14.9)	605 (34.2)	85.37	<0.001	700 (29.1)
Availability of purchase links ^c^	42 (6.6)	294 (16.6)	39.57	<0.001	336 (14.0)
Use of trending hashtags or topics	480 (57.0)	1277 (61.9)	5.92	0.015	1757 (60.5)

^a^ Only the numbers and percentages of posts that used these strategies were recorded. ^b^ Posts presented as text and images were excluded from the coding of this category; the total number for this category is *n* = 2597. ^c^ Pinduoduo was excluded from the coding of this category; the total number for this category is *n* = 2406.

**Table 3:** The effects of promotional strategies on audience feedback for healthy F&B (*n* = 639).

Category and Audience Feedback		Mean Ranks (Absence)	Mean Ranks (Presence)	*U*	*z*	*p*
**Use cue**						
	Likes	268.43	377.18	33,577.00	−7.44	<0.001
	Favorites	297.04	345.46	43,188.50	−3.31	0.001
	Comments	258.73	387.95	30,316.00	−8.84	<0.001
**Food Cue**						
	Likes	305.41	320.37	4750.50	−0.32	0.749
	Favorites	285.63	320.88	4434.00	−0.75	0.451
	Comments	334.53	319.63	4751.50	−0.32	0.750
**Chewing sounds ^a^**						
	Likes	271.69	379.93	29,596.00	−7.26	<0.001
	Favorites	289.00	350.77	36,362.50	−4.14	<0.001
	Comments	269.55	383.53	28,760.00	−7.64	<0.001
**Sensory description**						
	Likes	248.70	343.42	26,733.50	−5.60	<0.001
	Favorites	235.64	347.71	24,669.50	−6.62	<0.001
	Comments	255.11	341.31	27,747.00	−5.09	<0.001
**Family cue**						
	Likes	309.45	392.69	16,711.00	−3.79	<0.001
	Favorites	313.41	365.38	18,923.00	−2.37	0.018
	Comments	309.95	389.22	16,992.00	−3.61	<0.001
**Friend cue**						
	Likes	311.54	415.45	10,298.50	−3.89	<0.001
	Favorites	314.92	377.30	12,282.50	−2.34	0.020
	Comments	311.27	418.53	10,138.50	−4.02	<0.001
**Social proof**						
	Likes	324.64	262.93	11,444.50	−2.23	0.026
	Favorites	321.15	305.89	13,506.50	−0.55	0.582
	Comments	324.76	261.42	11,372.00	−2.29	0.022
**Nostalgia appeal**						
	Likes	318.80	415.00	1764.00	−1.46	0.143
	Favorites	319.21	382.06	2027.50	−0.96	0.339
	Comments	318.88	408.13	1819.00	−1.36	0.174
**Historical appeal**						
	Likes	320.29	294.07	2030.50	−0.37	0.709
	Favorites	319.59	356.86	1954.00	−0.53	0.595
	Comments	320.80	247.57	1705.00	−1.04	0.596
**Local cultural appeal**						
	Likes	310.12	389.01	16,839.00	−3.57	<0.001
	Favorites	314.98	355.11	19,551.50	−1.82	0.069
	Comments	309.56	392.96	16,523.00	−3.78	<0.001
**Quality description**						
	Likes	321.17	311.58	21,222.00	−0.43	0.667
	Favorites	323.46	295.12	19,938.50	−1.27	0.204
	Comments	319.05	326.82	21,347.00	−0.35	0.728
**Health benefit statement**						
	Likes	336.36	228.61	17,422.50	−5.29	<0.001
	Favorites	331.77	254.26	19,910.00	−3.81	<0.001
	Comments	334.78	237.42	18,276.50	−4.78	<0.001
**Nutritional statement**						
	Likes	322.14	284.19	9565.00	−1.20	0.231
	Favorites	319.14	334.46	10,333.50	−0.48	0.629
	Comments	322.23	282.71	9511.50	−1.25	0.212
**Price information**						
	Likes	325.42	278.59	17,841.00	−2.05	0.040
	Favorites	328.92	251.86	15,862.50	−3.38	0.001
	Comments	322.97	297.32	19,227.00	−1.12	0.261
**Discount information**						
	Likes	321.62	235.42	2747.00	−1.60	0.109
	Favorites	321.36	248.92	2909.00	−1.35	0.178
	Comments	321.67	232.96	2717.50	−1.65	0.099
**Brand visibility**						
	Likes	322.32	306.74	24,580.00	−0.76	0.448
	Favorites	325.37	289.27	22,921.00	−1.76	0.079
	Comments	320.75	315.69	25,431.00	−0.25	0.805
**Availability of purchase links**						
	Likes	321.88	293.25	11,413.50	−0.97	0.331
	Favorites	323.63	268.39	10,369.50	−1.87	0.061
	Comments	320.01	319.89	12,532.50	−0.004	0.997
**Use of trending hashtags or topics**						
	Likes	365.25	302.12	33,258.00	−3.90	<0.001
	Favorites	348.49	308.74	36,292.50	−2.45	0.014
	Comments	369.12	300.59	32,559.00	−4.23	<0.001

^a^ Posts presented as text and images were excluded from the coding of this category; therefore, *n* = 623.

**Table 4:** The effects of promotional strategies on audience feedback for unhealthy F&B (*n* = 1767).

Category and Audience Feedback		Mean Ranks (Absence)	Mean Ranks (Presence)	*U*	*z*	*p*
**Use cue**						
	Likes	719.27	982.93	256,758.50	−10.52	<0.001
	Favorites	826.25	918.68	327,686.00	−3.69	<0.001
	Comments	679.40	1006.87	230,324.00	−13.06	<0.001
**Food Cue**						
	Likes	679.90	885.75	10,078.50	−1.56	0.120
	Favorites	790.80	884.80	11,742.00	−0.710	0.447
	Comments	679.37	885.75	10,070.50	−1.56	0.119
**Chewing sounds ^a^**						
	Likes	759.64	967.71	288,107.50	−8.54	<0.001
	Favorites	820.40	920.29	334,771.00	−4.10	<0.001
	Comments	733.10	988.42	267,728.00	−10.48	<0.001
**Sensory description**						
	Likes	687.75	934.91	183,912.50	−8.24	<0.001
	Favorites	689.29	934.52	184,472.00	−8.17	<0.001
	Comments	697.58	932.37	187,488.00	−7.82	<0.001
**Family cue**						
	Likes	861.08	1049.43	131,273.50	−5.07	<0.001
	Favorites	862.18	1041.53	132,970.50	−4.83	<0.001
	Comments	864.40	1025.47	136,425.00	−4.34	<0.001
**Friend cue**						
	Likes	858.17	1048.35	143,795.50	−5.37	<0.001
	Favorites	864.21	1009.90	153,024.00	−4.11	<0.001
	Comments	868.29	983.93	159,257.00	−3.26	0.001
**Social proof**						
	Likes	877.16	983.21	82,911.00	−2.15	0.032
	Favorites	877.78	974.24	83,933.50	−1.95	0.051
	Comments	877.55	977.46	83,566.00	−2.02	0.043
**Nostalgia appeal**						
	Likes	880.96	949.88	60,732.00	−1.17	0.243
	Favorites	879.33	985.21	57,976.50	−1.79	0.073
	Comments	882.71	911.92	63,693.50	−0.49	0.621
**Historical appeal**						
	Likes	883.57	937.25	11,525.50	−0.39	0.695
	Favorites	883.04	1004.00	10,591.00	−0.88	0.377
	Comments	883.87	900.29	12,043.00	−0.12	0.905
**Local cultural appeal**						
	Likes	890.43	853.79	216,468.50	−1.15	0.251
	Favorites	905.67	782.13	194,256.00	−3.87	<0.001
	Comments	880.93	898.43	221,361.50	−0.55	0.583
**Quality description**						
	Likes	881.75	909.71	111,724.00	−0.63	0.531
	Favorites	888.94	827.50	107,351.50	−1.38	0.169
	Comments	880.36	925.67	109,457.50	−1.01	0.310
**Health benefit statement**						
	Likes	886.25	813.95	43,227.00	−1.03	0.301
	Favorites	885.30	843.62	44,859.00	−0.60	0.551
	Comments	886.37	810.24	43,023.00	−1.09	0.276
**Nutritional statement**						
	Likes	886.69	828.74	64,554.00	−1.00	0.315
	Favorites	884.73	869.10	67,863.00	−0.27	0.787
	Comments	884.74	868.77	67,836.00	−0.28	0.782
**Price information**						
	Likes	917.17	749.23	200,406.00	−5.51	<0.001
	Favorites	930.92	693.37	180,912.50	−7.79	<0.001
	Comments	911.40	772.66	208,584.50	−4.55	<0.001
**Discount information**						
	Likes	895.66	588.22	37,133.00	−4.84	<0.001
	Favorites	896.34	570.92	35,973.50	−5.12	<0.001
	Comments	894.81	609.81	38,579.00	−4.48	<0.001
**Brand visibility**						
	Likes	903.10	847.32	329,316.50	−2.18	0.029
	Favorites	903.98	845.63	328,291.00	−2.28	0.023
	Comments	904.50	844.62	327,682.50	−2.34	0.019
**Availability of purchase links**						
	Likes	901.75	795.05	190,381.00	−3.27	0.001
	Favorites	910.87	749.38	176,951.50	−4.96	<0.001
	Comments	898.79	809.91	194,750.00	−2.73	0.006
**Use of trending hashtags or topics**						
	Likes	931.40	864.13	300,202.50	−2.53	0.011
	Favorites	895.78	879.06	318,794.00	−0.63	0.530
	Comments	960.77	851.81	284,869.50	−4.10	<0.001

^a^ Posts presented as text and images were excluded from the coding of this category; therefore, *n* = 1752.

## Data Availability

The present data are available via https://doi.org/10.6084/m9.figshare.24298228 (accessed on 12 October 2023).
